# Application of BASNEF model in students training regarding cutaneous leishmaniasis prevention behaviors: a school-based quasi experimental study

**DOI:** 10.1186/s12879-021-06874-2

**Published:** 2021-11-17

**Authors:** Gholamreza Alizadeh, Hossein Shahnazi, Akbar Hassanzadeh

**Affiliations:** 1 School of Health, Isfahan University of Medical Sciences, Isfahan, Iran; 2 Department of Health Education and Health Promotion, School of Health, Isfahan University of Medical Sciences, Isfahan, Iran; 3 Department of Epidemiology and Biostatistics, Isfahan University of Medical Sciences, Isfahan, Iran

**Keywords:** Education, Student, Cutaneous leishmaniasis (CL), Prevention

## Abstract

**Background:**

Cutaneous leishmaniasis (CL) is endemic in 98 countries, and 350 million people are at risk of the disease worldwide. In endemic areas, conducting educational interventions is necessary to change preventive behaviors of CL. This study aimed to investigate the effect of an educational intervention based on the BASNEF model on CL preventive behavior in students.

**Methods:**

The present quasi-experimental study examined 80 students living in endemic areas of leishmaniasis in Isfahan province, Iran based on the BASNEF model. The required data were collected twice before and two months after the educational intervention based on a questionnaire whose validity and reliability had been already proven in other studies. The intervention was performed in three educational sessions for the students in the intervention group and 1 educational session for teachers and parents. Data were analyzed by SPSS (VER26) using the chi-square test, independent t-test, analysis of covariance (ANCOVA), and Paired t-test.

**Results:**

After intervention, the mean scores of Knowledge (P < 0.001), attitude (P = 0.02), subjective norms (P = 0.04), behavioral intention (P < 0.001), and behavior (P = 0.02) indicated significant differences between the intervention and control groups, but an increase in mean scores of enabling factors was not significant (P = 0. 93).

**Conclusions:**

Providing students with the educational intervention based on the BASNEF model improve their ability to the extent that they transmit these educations to their family members, which would be effective in preventing and controlling CL in leishmaniasis-prone areas.

***Trial registration*:**

Name: Iranian Registry of Clinical Trials. Registration number: IRCT20201024049131N1. Registration date: 2020–11-20. Registration timing: prospective.

## Background

Cutaneous Leishmaniasis (CL) is a skin infection that is still a major global health problem, especially in tropical and subtropical countries. CL, which is considered a neglected disease, is becoming more prevalent worldwide [1]. The disease is endemic in more than 98 countries [2], and 12 million people are infected with the disease worldwide, 350 million people are at risk [3–7], and every year, 1.5 million new cases of CL [5, 8] and 20,000 to 40,000 deaths due to this disease occur. [9, 10].

Iran is among the first ten countries in the world based on the number of cases [11]. According to WHO's report in 2017, more than 95% of new cases of CL occurred in Afghanistan, Algeria, Brazil, Colombia, Iran, Iraq, and Syria. [6, 12].

In Iran, CL is an important disease that is endemic in 18 provinces [13].In this country, more than 22,000 cases of leishmaniasis are being annually reported 80% of which are zoonotic CL [14]; however, the true number of infected cases is always 4 to 5 times more than the reported and recorded cases because of fear of treatment, and spontaneous improvement in patients, [15].

Comparison of statistics of CL in Iranian provinces indicates that it has the highest prevalence in Isfahan, Fars, and Khorasan provinces [16].For example, Jarghouyeh located in the east of Isfahan is among this province regions in which CL is highly prevalent [17].

This disease can cause many problems for patients, including psychological consequences due to prolonged wound period, the development of undesirable scars on the face, the possibility of secondary infections, the high cost of treatment for the society, the long treatment period, and side effects of treatment with existing drugs [18].

Researchers' failure to develop vaccines for CL and its high prevalence have made health education the top priority of WHO [19].

Numerous studies have also emphasized the importance of health education and public participation in the prevention of CL [2, 20]. Many researchers have suggested other disease control and prevention programs such as vaccine and drug production, environmental improvement, the extermination of mice, and poison spraying along with health education programs [7].

A major contributing factor to the development of this disease is that most people living in endemic areas don’t have enough knowledge about the way in which the disease transmission is prevented. Various studies have found that the public’s knowledge about CL is low [21, 22].This is a serious alarm because the necessary and correct information is the first and the most fundamental step towards any proper behavior [22].

The results of some studies suggest that instead of authentic resources, people obtain their information from family members, neighbors, and friends who are likely to convey incomplete information and misconceptions to people in society. [3, 23].

Attitude and beliefs of people living in endemic regions of CL need to be corrected or changed. For example, some people wrongly believe that mosquitoes transmitting CL are only present in regions and houses in which dogs and sheep are kept, suitable emollient creams and perfumes can prevent CL, and luck and God's wrath play roles in developing CL, etc. [24].

A very effective factor, which helps students living in endemic areas of leishmaniasis pursue CL preventive behaviors, is how much family, friends, classmates, health workers, principals, teachers, and educators take care to perform such behaviors. Family, friends, classmates, and teachers can play a major role in the process of behavior change in students.

A barrier to CL prevention behaviors is that even if people are encouraged to perform the behavior by education, some environmental limitations, such as lack of preventive tools, including proper netting and mosquito nets, insect repellent pen, etc. can prevent them from adopting appropriate behavior. [25].

Young groups and 10 to 15-year-old students are the most vulnerable groups to CL in the endemic areas. [26] In a study in Morocco, the children's face lesions were more than adults’ [27].

Given that students are the most vulnerable group to this disease and the most accessible group who can, by education, improve knowledge, attitude, and ultimately health behaviors of families, they were selected as the target group of the current study.

The selection of a model or theory is the most important measure taken in educational planning. A model or theory should be based on circumstances, problem, and alignment, and the quality of model/theory to be efficient and convergent with the purpose of the education program [28].

**Fig. 1:**
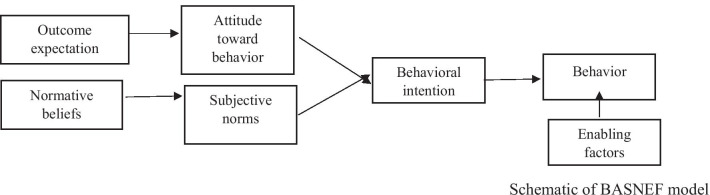
Schematic of BASNEF model

Since that the above-mentioned factors relating to CL preventive behaviors include:attitude, subjective norm, enabling factors, and behavioral intention, the researchers came to the conclusion that a suitable educational intervention can be designed and implemented to teach students to adopt CL preventive behaviors using the BASNEF model. (Fig. 1) [23].

Therefore, the present study aimed to investigate the effect of educational intervention based on the BASNEF model on CL prevention behaviors in male first-grade high school students in the east Isfahan.

## Methods

### Study design and sampling

The present quasi-experimental study examined 84 male first-grade high school students living in the eastern Isfahan in Iran, as an endemic region of leishmaniasis in Isfahan province, from January 2021 to May 2021. Multi-stage Random sampling was performed so that two cities (Mohammadabad and Hosseinabad with similar demographic variables and high incidence of CL) were first selected from cities located in Eastern Isfahan. Then, one of these two cities was randomly chosen as the city from which the students in the intervention group were drawn out and the other city was regarded as the city whose students were supposed to be included in the control group. After that, one school was randomly selected from each city and according to the list of students, 42 students were systematically chosen from the students of each school to be assigned to each group. The number of samples was calculated to be at least 70 by considering a 95% confidence level, 80% test power, and 10% probability of attrition [29].

The inclusion criteria were as follows: Studying at the first-grade high school, and the consent of students or their parents to participate in the study.

Individuals, who did not complete the questionnaires and educational sessions were excluded from the study.

### Data gathering tool

In this study, to collect data, the BASNEF model questionnaire, which was designed and validated by Ghodsi et al. [29] was used. The questionnaire included the following sections:

1. Demographic characteristics including the parents' education levels, the students’ or their family members’ status of being/not being infected with CL, the status of place of residence.
2. Knowledge items included four questions, which were designed as correct- incorrect and I do not know. The correct, I do not know, and incorrect options were scored 2, 1, and 0, respectively. Example: CL is transmitted by sandflies.
3. The section about the BASNEF model constructs, which included 32 questions, was as follows:

Six attitude-related items, including behavioral beliefs and outcome evaluation. For example: If I pursue CL prevention behaviors, I will not get it.

Eight subjective norms-related items, including normative beliefs and motivation to comply.

For example: How much do you try to do your activities based on your family, friends, classmates, health workers, teachers, and educators expectation?

Eight behavioral intention-related items. Example: I intend to use an insect repellent pen to protect myself from mosquito bites.

Five enabling factors-related items. Example: Existence of financial resources to buy wire net, insect repellent pen, insecticides, etc.

Five behavior-related items. Example: in the last three months, to what extent have you used insecticides to eliminate mosquitoes at home?

To answer the above items, the five-point Likert scale was used which were scored from 0 to 4. In each construct, a higher score indicated a better status. For the ease of comparison, in different sections of the questionnaire, all scores were reported out of 100.

AGFI = 0.9 and RMESA = 0.04 were obtained in the construct validity. The reliability of the questionnaire was confirmed using internal consistency (α = 0.864) [29].

### Educational intervention

The educational content was set by reviewing the existing literature (guidebooks, the prevention of Cutaneous Leishmaniasis by the Zoonosis Center for Disease Control of the Ministry of Health). In the intervention group, the educational intervention was conducted in three 60-min sessions in the form of lectures, group discussions, brainstorming, questions and answers, and practical demonstrations.

The first session included various educational methods such as lectures, showing photos and videos, and questions and answers on the agent, reservoir, and vectors of CL, gathering and resting places, and time of sandfly bites. Then, the role of body cover, using pen repellent, and insecticide spray on the prevention of bites was explained.

The second session discussed the most important common beliefs about CL in the region as well as their right and wrong nature, the way of changing misconceptions in families, and the importance of CL prevention behaviors among students.

In the third session, students became familiar with the facilitators and barriers to CL prevention behaviors and practiced overcoming strategies, appropriate window nets and mosquito netting were also directly shown to the students and their effective role in the prevention of bites was explained, and they practically learnt how to use insect repellent pen.

Table 1 summarizes the presented training based on the BASNEF model constructs.

**Table 1:** Summary of education based on the BASNEF model constructs

BASNEF model constructs	Cognitive-behavioral goals	Learning domain	Educational technique
Knowledge	Learning about the reservoir and vectors of the disease, resting place, and time of mosquito bites, the role of body cover, insect repellent pen, insecticide spray, and characteristics of window net and suitable mosquito netting for preventing CL	Cognitive	Lecture, question and answer, PowerPoint
Attitude	Discussing the most important common beliefs, their nature of being right or wrong common beliefs, the way of changing misconceptions, and the importance of CL prevention behaviors to prevent infection	Affective	Questions and answer, group discussion
Subjective norms	Familiarization of parents and teachers with CL and prevention methods to continuously transmit this information to students and improve social support from teachers and students' parents	Cognitive-affective	Questions and answer (group discussion
Enabling factors	Facilitators and barriers to performing preventive behaviors, increasing the skill of using a repellent pen and spray, and introducing appropriate nets and mosquito netting, and solutions to overcome barriers to CL prevention behaviors	Cognitive—psychomotor	Lecture, practical demonstration, role-playing

To attract the students' social support, a training session was designed for parents and teachers on CL prevention behavior and its importance. In this sessions, in addition to providing necessary training (e.g. about the reservoir and vector of CL, time of mosquito bites, the resting place of mosquito, the correct way of using insect repellent pen, the impact of insecticide spray, and features of appropriate mosquito netting), parents and teachers were recommended to appropriately support students to adopt CL prevention behaviors.

The questionnaires were completed by students of the two groups before the training sessions and two months after the end of the sessions.

### Data analysis

The completed questionnaires before and after the intervention were inserted into SPSS (Ver. 26).

To compare demographic variables between the two groups, the Chi-square and Independent t-test were used. Before the intervention, the mean scores of knowledge and constructs of the BASNEF model between the two groups were compared through the Independent t-test, while after the intervention, scores of knowledge and constructs of the BASNEF model between the two groups were compared by using analysis of covariance(ANCOVA). The Paired t-test was also employed to compare mean scores of knowledge and constructs of the BASNEF model in each group before and after the intervention.

## Results

In the present study, the mean age of students in the intervention and control groups was 14.3 ± 1.03 and 14.1 ± 0.8 years, respectively. The independent t-test indicated that there was not any significant difference between the two groups in terms of the mean age (P = 0.28) and the number of family members (P = 0.34).

The Chi-square test, on the other hand, indicated that there was not any significant difference between the two groups in terms of the frequency distribution of CL status (P = 0.20), the status of CL in the family (P = 0.65), as well as the frequency distribution of the residence place (P = 0.26), and the residence status (P = 0.47). Also, the Mann–Whitney test showed that there was not any significant difference between the two groups in terms of students' education grade (P = 0.25), fathers' education level (P = 0.20), and mothers' education level (P = 0.08) (Table 2).

**Table 2:** Comparison of demographic characteristics between experimental and control groups

Variable	Experimental group		Control group		P-value
	No	Percentage	No	Percentage	
CL status in students					
Currently suffering from CL	1	2.5	0	0	0.20
Previously infected with cutaneous leishmaniasis	13	32.5	8	20	
No history of CL	26	65	32	80	
CL status in the family					
Currently suffering from CL	3	7.5	2	5	0.65
Previously infected with CL	18	45	15	37.5	
No history of CL	19	47.5	23	57.5	
Place of residence					
Suburbs	26	65	21	52.5	0.26
Downtown	14	35	19	47.5	
Residence place status					
Newly constructed	26	65	29	72.5	0.47
Old	14	35	11	27.5	
Educational grade					
Grade 7	10	25	12	30	0.25
Grade 8	12	30	16	40	
Grade 9	18	45	12	30	
Fathers' educational level					
Illiterate	4	10	7	17.5	0.20
Primary and secondary school	19	47.5	22	55	
High school diploma	13	32.5	6	15	
Academic	4	10	5	12.5	
Mothers' educational level					
Illiterate	4	10	8	20	0.08
Primary and secondary school	19	47.5	22	55	
High school diploma	11	27.5	6	15	
Academic	6	15	4	10	

Moreover, the ANCOVA test revealed that after the educational intervention, the mean scores of knowledge (P < 0.001), attitude (P = 0.02), behavioral intention (P < 0.001), behavior (P = 0.02), and subjective norms (P = 0.04) were significantly higher in the intervention group than the control group, but there was not any significant difference between mean scores of enabling factors of the two groups (P = 0.93). (Table 3).

**Table 3:** The Comparison of mean scores of BASNEF model constructs between the intervention and control groups before and two months after educational intervention (Between-group comparison)

Variable		Intervention group Mean (SD)	Control group Mean (SD)	P-value
Knowledge	Before intervention	61.9 (20.4)	63.6 (21.7)	0.71*
	After intervention	86.2 (13.8)	62.4 (20.4)	< 0.001**
Attitude	Before intervention	72.8 (18.9)	73.4 (19.6)	0.90*
	After intervention	81.6 (15.6)	72.9 (18.5)	0.02**
Behavioral intention	Before intervention	67.5 (19.3)	70.6 (15.9)	0.45*
	After intervention	82.7 (10.9)	70.9 (14.4)	< 0.001**
Behavior	Before intervention	54.1 (25.6)	56.8 (21.04)	0.61*
	After intervention	66.2 (18.5)	55.3 (23.6)	0.02**
Subjective norms	Before intervention	71.8 (19.1)	73.8 (18.1)	0.63*
	After intervention	77.4 (15.3)	70.8 (17.1)	0.04**
Enabling factors	Before intervention	68.4 (19.8)	69.6 (20.3)	0.79*
	After intervention	70.4 (13.3)	69.7 (16.2)	0.93**

*Independent T-test

** ANCOVA T-test

The paired t-test indicated that in the intervention group, the mean scores of knowledge (P < 0.001), attitude (P = 0.002), behavioral intention (P < 0.001), behavior (P = 0.01), and subjective norms (P = 0.04) were significantly higher after the intervention compared with before the intervention, but the mean scores of enabling factors after the intervention were not significantly different from those obtained before the intervention (P = 0.53). The same test showed that in the control group, the mean scores of BASNEF model constructs after the intervention were not significantly different from those obtained after the educational intervention (P > 0.05) (Table 4).

**Table 4:** Comparison of mean scores of the BASNEF model constructs in each of the intervention and control groups before and two months after the educational intervention (Within-group comparison)

Variable		Before intervention Mean (SD)	After intervention Mean (SD)	P-value*
Knowledge	Intervention group	61.9 (20.4)	86.2 (13.8)	< 0.001
	Control group	63.6 (21.7)	62.4 (20.4)	0.77
Attitude	Intervention group	72.8 (18.9)	81.6 (15.6)	0.002
	Control group	73.4 (19.6)	72.9 (18.5)	0.93
Behavioral intention	Intervention group	67.5 (19.3)	82.7 (10.9)	< 0.001
	Control group	70.6 (15.9)	70.9 (14.4)	0.92
Behavior	Intervention group	54.1 (25.6)	66.2 (18.5)	0.01
	Control group	56.8 (21.04)	55.3 (23.6)	0.75
Subjective norms	Intervention group	71.8 (19.1)	77.4 (15.3)	0.04
	Control group	73.8 (18.1)	70.8 (17.1)	0.36
Enabling factors	Intervention group	68.4 (19.8)	70.4 (13.3)	0.53
	Control group	69.6 (20.3)	69.7 (16.2)	0.90

*Paired T-test

## Discussion

The present study aimed to determine the impact of educational interventions on the improvement of CL prevention behaviors among the male first-grade high school students in east Isfahan (an endemic region of CL).

In the study, the intervention and control groups were homogeneous in terms of demographic characteristics. The mean scores of knowledge and constructs of the BASNEF model were not significantly different between the intervention and control groups before the educational intervention, indicating a minimum effect of confounding variables on the research results.

The results further indicated a significant increase in the mean knowledge score of the intervention group after intervention, while there was not any significant change in the control group, indicating the effect of education on raising students' knowledge about CL.

The necessary and correct information about the disease is the most basic step towards appropriate behavior. Some studies similarly suggest that educational intervention based on the BASNEF model is more effective in raising knowledge than classic education [30, 31].

The reason for the intervention group’s higher attitude mean score after the intervention was student's participation in the educational sessions, the management of the educational sessions based on workshop method, and learning through problem solving, which not only did not inject the students with educational content, but also allowed them to express their ideas and beliefs. In fact, expressing idea and seeing other people's reactions is a powerful way to change attitudes confirmed in other studies [32].

In studies by Shabidar et al. and Azideh maab et al., which were both on the effect of interventions on attitude change, the educational intervention did not significantly change the attitude scores of the participants [33, 34]. The difference in the effect of education on attitudes in different studies might be rooted in the fact that compared with knowledge, attitude and behavior are often affected by a set of multiple environmental and social factors; hence, education and information alone cannot correct them. Therefore, increasing knowledge alone cannot improve attitude.

Based on the results of the present study, subjective norms, such as parents, friends, classmates, and teachers play important roles in providing appropriate social support for students. Therefore, in addition to providing students with educational interventions, influential individuals in the students' life should be identified and become directly and indirectly involved in educational interventions because important others (subjective norms) could be very effective in behavioral changes [35, 36].

In the present study, the mean scores of the behavioral intention of the students in the intervention group increased after educational intervention. Behavioral intention is an index showing a person's readiness to perform a particular behavior and is an immediate predictor of behavior. Intentions contain motivational factors that are resulted from attitudes and subjective norms; hence, they can lead to behavior. On the other hand, a positive attitude towards behavior alone cannot guarantee the performance of the behavior, but according to theories and models, attitudes affect the performance of behavior by affecting the behavioral intention. The subjective norms, or people who are important to a person, can also help the person in the behavioral intention [37].

An effective factor in performing CL prevention behaviors is the availability of a suitable insect repellent pen, spray, and mosquito netting. Given that these items are relatively expensive and most students were from either low- or medium-income families, they couldn't afford to buy these items despite having the appropriate knowledge and attitude towards the provision of these preventive items. Lack of supply of these items for free during the education by the local health center (some of these items had been distributed for free leading to expectations in the residents) was a reason for the significant increase in scores of enabling factors in the intervention group after the intervention.

Pardo's et al. study indicated that families with better economic status and higher income use more preventive means than other people; thus, the incidence of the disease was lower among them [38].

In studies by Sharifirad [39] and Mohebi [40], easy access and cost were the most important enabling factors. In contrast with the present study, in studies by Hazavei et al. [41], Arefi et al. [32], which were conducted based on the BASNEF model, the increase in mean scores of enabling factors was significant after the educational intervention.

The results indicated that the students' behavior mean scores significantly increased in the intervention group after the educational intervention, indicating the impact of the intervention based on the BASNEF model.

## Conclusion

Educational intervention based on the BASNEF model increased the knowledge and skills necessary for CL prevention behaviors. However, some barriers cannot be removed by educational interventions alone. These barriers include lack of money to buy the necessary equipment, the presence of rubble and construction debris in residential areas, and the low level of environmental health, etc.

Asking for other organizations’ help (municipalities, environment, housing, etc.) and charitable groups and non-governmental organizations, and attracting inter-sectoral collaboration are main solutions to solve the problem of CL in endemic regions. Therefore, it seems that a very suitable solution is to attract comprehensive support that should be provided by the national health care system to attract the participation of various groups and organizations to improve CL prevention behaviors among people living in endemic regions.

The use of a model base intervention to promote preventive behaviors from CL was among the strengths of the research; nonetheless, holding face-to-face educational sessions was confronted with limitation due to COVID-19 outbreak.

## Data Availability

The data that support the findings of this study are available from Deputy of research of Isfahan University of Medical Sciences but restrictions apply to the availability of these data, which were used under license for the current study, and so are not publicly available. Data are however available from the authors upon reasonable request and with permission of Isfahan University of Medical Sciences.

## Abbreviation

CL: Cutaneous leishmaniasis
